# Molecular characterization of rotavirus group A strains circulating prior to vaccine introduction in rural coastal Kenya, 2002-2013

**DOI:** 10.12688/wellcomeopenres.14908.2

**Published:** 2019-05-15

**Authors:** Betty E. Owor, Mike J. Mwanga, Regina Njeru, Robert Mugo, Mwanajuma Ngama, Grieven P. Otieno, D.J. Nokes, C.N. Agoti

**Affiliations:** 1 Epidemiology and Demography, KEMRI Wellcome Trust Research Program, Kilifi, Kilifi, 80108, Kenya; 2 School of Life Sciences and Zeeman Institute for Systems Biology and Infectious Disease Epidemiology, Warwick University, Coventry, Coventry, CV4 7AL, Kenya; 3 School of Health and Human Sciences, Pwani University, Kilifi, Kilifi, 80108, Kenya

**Keywords:** Rotavirus, epidemiology, genotype, strains, diversity

## Abstract

**Background: **Kenya introduced the monovalent Rotarix® rotavirus group A (RVA) vaccine nationally in mid-2014.  Long-term surveillance data is important prior to wide-scale vaccine use to assess the impact on disease and to investigate the occurrence of heterotypic strains arising through immune selection. This report presents baseline data on RVA genotype circulation patterns and intra-genotype genetic diversity over a 7-year period in the pre-vaccine era in Kilifi, Kenya, from 2002 to 2004 and from 2010 to 2013.

**Methods: **A total of 745 RVA strains identified in children admitted with acute gastroenteritis to a referral hospital in Coastal Kenya, were sequenced using the di-deoxy sequencing method in the VP4 and VP7 genomic segments (encoding P and G proteins, respectively). Sequencing successfully generated 569 (76%) and 572 (77%) consensus sequences for the VP4 and VP7 genes respectively. G and P genotypes were determined by use of BLAST and the online RotaC v2 RVA classification tool.

**Results: **The most common GP combination was G1P[8] (51%), similar to the Rotarix® strain, followed by G9P[8] (15%) , G8P[4] (14%) and G2P[4] (5%).  Unusual GP combinations—G1P[4], G2P[8], G3P[4,6], G8P[8,14], and G12P[4,6,8]—were observed at frequencies of <5%. Phylogenetic analysis showed that the infections were caused by both locally persistent strains as evidenced by divergence of local strains occurring over multiple seasons from the global ones, and newly introduced strains, which were closely related to global strains. The circulating RVA diversity showed temporal fluctuations both season by season and over the longer-term. None of the unusual strains increased in frequency over the observation period.

**Conclusions: **The circulating RVA diversity showed temporal fluctuations with several unusual strains recorded, which rarely caused major outbreaks.  These data will be useful in interpreting genotype patterns observed in the region during the vaccine era.

## Introduction

Rotavirus group A (RVA) infection is a leading cause of childhood severe dehydrating acute diarrhoea, which can lead to death ^1^. The 2016 estimates show that, annually, RVA is responsible for 128,500 deaths globally, with the highest burden occurring in sub-Saharan Africa and South-East Asia countries ^2^. In 2009, the World Health Organization (WHO) recommended the inclusion of either of the two licensed RVA vaccines (Rotarix® and RotaTeq®) into national immunization programmes (NIPs) of all countries to curb RVA associated disease burden ^3^. Kenya introduced the monovalent Rotarix® vaccine (based on the G1P[8] strain) into its NIP in July 2014 ^4^.

In Africa, the introduction of the Rotarix® vaccine into the NIPs of several countries has been associated with a marked reduction in hospitalization caused by RVA infection ^5, 6^. For instance in Malawi, Burkina Faso and Tanzania, the vaccine effectiveness against hospitalization was estimated at 62%, 58% and 53%, respectively ^7^. However, this effectiveness is lower than that observed in developed countries; for example in Belgium, vaccine effectiveness of Rotarix vaccine was estimated at 90% ^8^. Furthermore, concerns remain that in time, given the high diversity of RVA strains, vaccine immunity escape variants could emerge which may undermine the gains from the vaccination programmes ^9^. Such a scenario was observed in Japan where, a G8P[8] RVA strain appeared to emerge and was found in up to 66% (53/80) of children with acute gastroenteritis disease attending one pediatric clinic in Shizuoka Prefecture in February – July 2017 ^10^. Similarly, the predominance of non-vaccine type G2P[4] strains was observed in Rotarix® vaccinated populations of Belgium ^11^ and Brazil ^12^. Although the rise was not linked to pressure induced by the vaccine, concerns still remain on the overall effect of the vaccine on circulating vaccine heterotypic strains.

The rotavirus genome is comprised of 11 segments of double-stranded RNA, which encode 11 proteins (VP1-4, VP6, VP7, NSP1-5) and sometimes 12 (NSP6). The VP7 and VP4 proteins independently elicit neutralizing antibodies and specify the G (glycoprotein) and P (protease-sensitive) genotypes, respectively ^13^. Molecular characterization of the VP7 and VP4 proteins encoding regions is commonly used to investigate local and global RVA molecular epidemiology and is the basis of the dual genotype classification of this virus ^14^. Up to 36 different G and 51 P RVA genotypes have been identified worldwide in humans and animals ^15^. Globally, G1P[8], G2P[4], G3P[8], G4P[8], G9P[8] have been identified as the most common genotypes (in decreasing order) while G12P[6] and G12P[8] have recently been reported as emerging genotypes. The distribution of the genotypes can vary considerably from region to region and from one season to the next ^16, 17^. While globally dominant genotypes are similarly dominant in Africa ^18^, understanding of their local natural seasonal fluctuations and intra-genotype diversity in the pre-vaccine introduction era is incomplete despite importance to vaccine impact evaluation.

The current study presents molecular analysis of historical RVA strains from coastal Kenya detected between 2002–2004, reported in Nokes *et al.* ^19^, which we refer to as phase I, together with more recent RVA strains detected between 2010–2013, referred to as phase II. We present findings from partial sequence analysis of these longitudinally collected RVA strains identified at the Kilifi County Hospital (KCH), Kilifi, Kenya, and phylogenetically compare these with those deposited in public databases isolated across the globe. The GP typing of phase I strains was previously performed by nested multiplex PCR using genotype-specific VP7 and VP4 primers ^19^. We utilize these extensive sequence data to illuminate on local RVA genotype circulation characteristics and provide baseline information on natural patterns of RVA genotype diversity in coastal Kenya prior to vaccine introduction.

## Methods

### RVA surveillance in Kilifi County Hospital

RVA surveillance in KCH reported in this analysis was conducted from January 2002 to December 2004 (phase I), and from January 2010 to December 2013 (phase II). Study subject recruitment criteria and sample collection methods are as previously described ^19^. The study targeted children aged less than 13 years admitted with acute diarrhoea defined as three or more watery stools passed during a 24-hour period ^20^. The KEMRI Scientific and Ethics Review Unit (SERU) in Kenya approved the study protocol (#SERU 3049).

### Detection of RVA

Stool samples were screened for RVA using an enzyme immunoassay (EIA) kit, marketed under two different names in the two periods: IDEIA (DAKO Rotavirus IDEIA ^TM^, Oxoid, Ely, United Kingdom) in phase I and ProSpect ^TM^ (Oxoid, Basingstoke UK) in phase II, following the manufacturer's instructions.

### Partial sequencing of RVA positive samples in VP4 and VP7 segments

Sequencing was conducted on 272 (46%) of 558 positive samples detected in phase I, and all positive samples identified in phase II (n=473). The phase I samples for sequencing were selected to represent common RVA genotypes (>70%) observed throughout the surveillance period from each year. Partial fragments of the VP4 and VP7 genes, were amplified in a One-Step reverse transcriptase PCR reaction using the following primer pairs: VP4F, 5’-TATGCTCCAGTNAATTGG-3’, VP4R 5’-ATTGCATTTCTTTCCATAATG-3’, VP7F, 5’-ATGTATGGTATTGAATATACCAC-3’, VP7R 5’–-AACTTGCCACCATTTTTTCC-3’, as previously described by Simmonds *et al.* ^21 ^and Gomara *et al.* ^22^. To confirm successful amplification of the targeted genomic area the products were checked (VP7, 881 bp; VP4, 660 bp) by electrophoresis in a 2% agarose gel. Products of samples that showed presence of the expected band size on gels were purified using GFX DNA purification kit (GFX-Amersham, UK) following the manufacturer’s instructions. These were then sequenced using Big Dye Terminator 3.1 (Applied Biosystems, Foster City, California, USA) chemistry and the same primers as in PCR amplification on an ABI Prism 3130xl Genetic Analyser (Applied Biosystems, Foster City, California, USA).

### RVA genotyping and sequence analysis

During phase I surveillance, RVA genotypes were determined by nested multiplex PCR using genotype-specific VP7 and VP4 primers, enabling identification of mixed genotypes ^19^. In phase II, genotypes were determined by sequence of the VP7 and VP4 genes, which was limited in calling mixed infections. The sequence reads were assembled into contigs using Sequencher version 5.4.6 (Gene Codes Corp Inc., Ann Arbor, MI, USA). The nucleotide sequences were aligned using MAFFT version 7.222 ^23^ and visualized in Aliview version 1.8 and further trimmed to remove sequence overhangs, resulting in contigs of lengths between 480-660 bp (coordinates; 184-748) covering ~23% of the VP4 gene, and 486-854 bp (coordinates; 460-824 of the VP7 gene) covering ~67% of the VP7 gene. G and P genotypes were determined using NCBI BLAST for sequences <500 bp (n=13 for VP4, n=5 for VP7) and the RotaC version 2.0 classification tool ^24^ for sequences >500 bp. MEGA v7.0.26 was used to select the best maximum likelihood evolution models based on the Bayesian Information Criterion ^25^ ( Supplementary Table 1) and reconstruction of maximum likelihood phylogenetic trees with 500 bootstrap replicates. Global contemporaneous sequences (2002–2013) (accession numbers in Supplementary File 1, lists 1 and 2) together with the Rotarix® vaccine strain sequences were retrieved from GenBank database and phylogenetically compared with the local sequences. Duplicate sequences from the same country and non-overlapping sequences were removed. Clusters were identified based on high bootstrap values of >70% and high nt sequence similarity of >98%. Nucleotide and amino acid pairwise distances between the sequences were determined in MEGA v7.0.26. The trees were drawn to scale indicating nucleotide substitution rates per site.

## Results

The prevalence of the genotypes and their circulation patterns were inferred using all the data collected in 2002–2004 (phase I) surveillance period irrespective of whether they were selected here for sequencing and all data collected between 2010–2013 (phase II). Data are available under restriction on Harvard Dataverse ^26^.

### RVA prevalence in KCH pediatric diarrhoea admissions

Over the 7-year surveillance period, a total of 3,779 stool samples were screened for RVA using EIA, of which 27.3% (n=1,031) tested positive. In phase I, the prevalence of RVA in the study population was 27.4% (n=558) while in phase II the prevalence was 27.2% (n=473) ( Table 1). Among the selected samples for sequencing was successful for 569 (76%) and 572 (77%) samples for the VP4 and VP7 segments, respectively ( Table 1).

**Table 1.** A summary of diarrhoea cases, the number of samples tested, the proportion of RVA cases observed in the entire surveillance period and the number of samples sequenced from each phase from childhood admissions to KCH, Kenya, between 2002–2004 and 2010–2013.

Period	Admissions, n	Diarrhoea, n	Samples tested, n	RVA cases, n	Proportion, %	Sequenced, n (%)	Successfully assembled, n (%)	
							VP4	VP7
2002–2004 (Phase I)	15347	3296	2039	558	27.2	272 (48)	192 (71)	218 (80)
2010–2013 (Phase II)	11579	2260	1740	473	27.4	473 (100)	377 (80)	354 (75)
Total	26926	5556	3779	1031	27.3	745 (72%)	569 (76)	572 (77)

### RVA genotypes in the study populations

**Figure 1.**
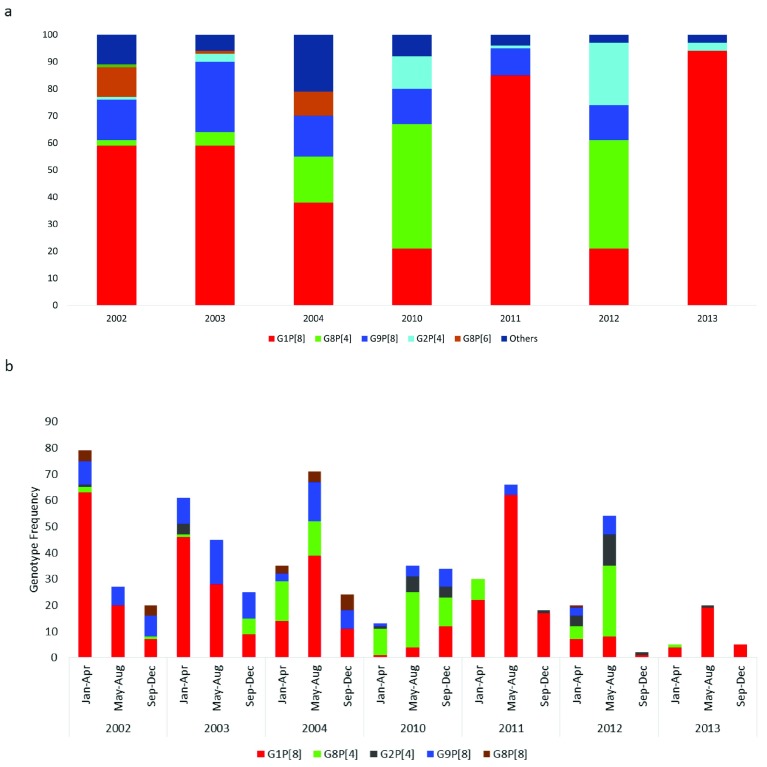
Temporal distribution of RVA genotypes from positive RVA cases isolated from Kilifi County Hospital from 2002–2004 and 2010–2013. ( **A**) Annual proportions of the common genotypes. ( **B**) The 4-month frequency of the commonly occurring genotypes. The colors represent the GP combinations as indicated on the legend of the plots. Genotypes that fall in the “Others” category in ( **A**) represents those that occurred in low frequency <5%: G8P[8,14], G1P[4,6], G9P[4,6], G2P[8], G12P[4,6,8], G3P[4,6], G10P[8].

The G genotypes identified in patients admitted at the KCH were G1-G3, G8-G10, G12, G29, while the P genotypes were P[4], P[6], P[8] and P[14] genotypes. Overall, G1P[8] was the dominant strain at 51% followed by G9P[8] (15%), G8P[4] (14%) and G2P[4] (5%) as shown in Table 2 and Supplementary Figure 1. Uncommon strains bearing G1P[4], G2P[8], G3P[4,6,8], G8P[6,8,14], G9P[4,6], G12P[4,6,8] were also detected, albeit in low frequency (<5%). The use of genotype-specific primers to identify RVA GP genotypes in phase I enabled the detection of mixed infections in 8.2% of the samples collected between 2002–2004; however, in phase II we used the sequencing approach to infer genotypes and this approach is not suited for detection of mixed infections. Additionally, among both phase I and II samples, 9.2% of these samples were genotyped for only one of the two genes due to failed in sequencing and/or contig assembly for the second gene ( Table 2). G1P[8] predominated in all the years of phase I, while in phase II, this genotype was dominant only in 2011 and 2013 ( Figure 1A, B). Whilst strain G8P[4] was observed in low frequency in the whole of phase I, it was observed as the most common strain in 2010 (46%) and 2012 (40%). Strain G9P[8] circulated in low proportions, and was observed in all the years except in 2013. Strain G2P[4] which was observed in low frequency in phase I (1.1%), was seen to increase in proportions in years 2010 (12%) and 2012 (23%). The rare strains, G1P[6], G2P[8] G8P[6] and G9P[6] were only observed in phase I, while the rare strains G3P[4,6], G12P[4,6,8], G10P[8] and G8P[14] were observed only in phase II. None of the rare strains observed in phase I became common in phase II.

**Table 2.** Frequency and proportions of RVA strains observed in Kilifi County Hospital between 2002–2004 and 2010–2013.

	Type	2002		2003		2004		2010		2011		2012		2013		Total	
		n	%	n	%	n	%	n	%	n	%	n	%	n	%	n	%
GP Genotypes	G1P[8]	90	(59)	79	(59)	63	(38)	19	(21)	101	(85)	16	(21)	28	(93)	396	51
	G9P[8]	23	(15)	36	(27)	25	(15)	12	(13)	12	(10)	10	(13)	0	(0)	118	15
	G8P[4]	3	(2)	7	(5)	28	(17)	42	(46)	0	(0)	30	(40)	0	(0)	110	14
	G2P[4]	1	(1)	4	(3)	0	(0)	11	(12)	1	(1)	17	(23)	1	(3)	35	5
	G8P[6]	17	(11)	1	(1)	15	(9)	0	(0)	0	(0)	0	(0)	0	(0)	33	4
	G8P[8]	8	(5)	1	(1)	13	(8)	0	(0)	0	(0)	2	(3)	0	(0)	24	3
	G1P[4]	0	(0)	0	(0)	10	(6)	3	(3)	0	(0)	0	(0)	0	(0)	13	2
	G9P[6]	3	(2)	4	(3)	5	(3)	0	(0)	0	(0)	0	(0)	0	(0)	12	2
	G1P[6]	3	(2)	0	(0)	6	(4)	0	(0)	0	(0)	0	(0)	0	(0)	9	1
	G9P[4]	0	(0)	2	(1)	1	(1)	2	(2)	0	(0)	0	(0)	0	(0)	5	1
	G2P[8]	4	(3)	1	(1)	1	(1)	0	(0)	0	(0)	0	(0)	0	(0)	6	1
	G3P[8]	0	(0)	0	(0)	0	(0)	0	(0)	0	(0)	0	(0)	1	(3)	1	0
	G12P[8]	0	(0)	0	(0)	0	(0)	1	(1)	3	(3)	0	(0)	0	(0)	4	1
	G12P[4]	0	(0)	0	(0)	0	(0)	1	(1)	1	(1)	0	(0)	0	(0)	2	0
	G12P[6]	0	(0)	0	(0)	0	(0)	0	(0)	1	(1)	0	(0)	0	(0)	1	0
	G8P[14]	0	(0)	0	(0)	0	(0)	1	(1)	0	(0)	0	(0)	0	(0)	1	0
	Total	152	(100)	135	(100)	167	(100)	92	(100)	119	(100)	75	(100)	30	(100)	770	100
Mixed & Non Typable	Mixed	7	(27)	24	(71)	26	(59)	0	(0)	0	(0)	0	(0)	0	(0)	57	55
	GNTP[4]	0	(0)	0	(0)	6	(14)	0	(0)	0	(0)	0	(0)	0	(0)	6	6
	GNTP[6]	2	(8)	0	(0)	3	(7)	0	(0)	0	(0)	0	(0)	0	(0)	5	5
	GNTP[8]	8	(31)	0	(0)	1	(2)	0	(0)	0	(0)	0	(0)	0	(0)	9	9
	G1PNT	5	(19)	5	(15)	2	(5)	0	(0)	0	(0)	0	(0)	0	(0)	12	12
	G8PNT	1	(4)	1	(3)	1	(2)	0	(0)	0	(0)	0	(0)	0	(0)	3	3
	G9PNT	2	(8)	2	(6)	2	(5)	0	(0)	0	(0)	0	(0)	0	(0)	6	6
	GNTPNT	1	(4)	2	(6)	3	(7)	0	(0)	0	(0)	0	(0)	0	(0)	6	6
	Total	26	100	34	100	44	100	0	0	0	0	0	0	0	0	104	100
	G1P[x]	0	(0)	0	(0)	0	(0)	0	(0)	7	(27)	2	(13)	7	(22)	16	16
Failed sequencing	G3P[x]	0	(0)	0	(0)	0	(0)	0	(0)	0	(0)	0	(0)	3	(9)	3	3
	G8P[x]	0	(0)	0	(0)	0	(0)	7	(26)	0	(0)	1	(6)	0	(0)	8	8
	G9P[x]	0	(0)	0	(0)	0	(0)	0	(0)	2	(8)	1	(6)	0	(0)	3	3
	G12P[x]	0	(0)	0	(0)	0	(0)	3	(11)	2	(8)	2	(13)	0	(0)	7	7
	G29P[x]	0	(0)	0	(0)	0	(0)	1	(4)	0	(0)	0	(0)	0	(0)	1	1
	GxP[8]	0	(0)	0	(0)	0	(0)	7	(26)	15	(58)	5	(31)	19	(59)	46	46
	GxP[4]	0	(0)	0	(0)	0	(0)	9	(33)	0	(0)	5	(31)	3	(9)	17	17
	Total	0	0	0	0	0	0	27	100	26	100	16	100	32	100	101	100

NT, non-typable; Gx and Px, undetermined G and P genotypes, respectively.

### Genetic relationship between RVA strains

**Figure 2.**
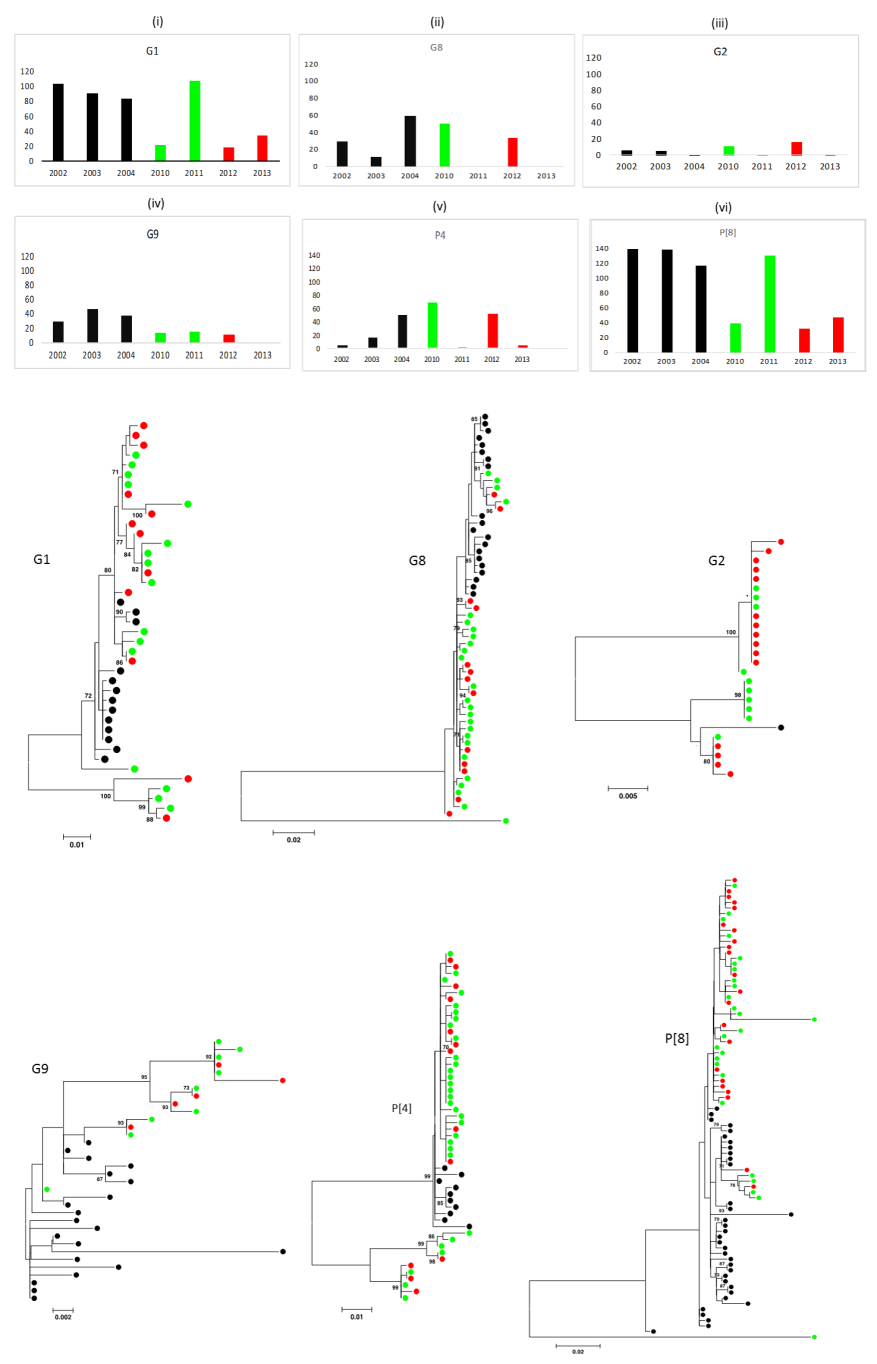
Maximum likelihood phylogenetic trees of G1, G2, G8, G9, P[4] and P[8] genotypes inferred in MEGA v7, with taxa stratified in 3 groups, black for 2002–2004, green for 2010–2011 and red for 2012–2013, from viruses detected in childhood diarrhea admissions to KCH, Kenya. The bar graphs represent frequency of the same genotypes between 2002–2004 and 2010–2013. Only bootstrap values ≥70% are shown. This figure excludes the infrequent genotypes G3, G10, G12, G29 and P[6] and P[14]. The scale bars indicate nucleotide substitutions per site.

Figure 2 shows the temporal frequency of genotypes G1, G2, G8, G9, P[4] and P[8] with their corresponding phylogenetic trees, while nucleotide pairwise difference within each genotype is shown in Supplementary Figure 2. The time period is split into 2002–2004, 2010–2011 and 2012–13 (shown by different colours) to facilitate temporal comparison. The G1 strains which were observed in all the years, formed clusters containing strains from both phase I and II, showing an overall sequence homology of >92% at the nucleotide level. Additionally, minor distinct clusters containing strains observed in phase II were also observed. The occurrence of G8 strains fluctuated with high prevalence observed in 2002, 2004 and 2010, and less prevalent in 2003 and 2009. Majority of the G8 strains showed high sequence homology of 96–100%, forming a common cluster including strains from both phases. However, a single sequence showed a decrease in homology up to 84% at nt level and formed distinct clusters. The infrequently occurring G2 strains, formed two distinct clusters, where one cluster contained strains observed in phase II while the other had strains from both phases. Nevertheless, a high sequence homology of >95% at the nucleotide level was observed within G2 strains. Such high sequence homology was also observed in G9 strains, which were observed in high frequencies in all epidemic years except 2013.

Phylogenetically, the P[8] strains showed a close association among themselves with sequence similarities of between 92–100% at nt level. Despite, the high homology, majority of the P[8] strains observed in phase I formed separate clusters from those observed in phase II. Unlike P[8] strains, P[4] strains occurred less frequently, with high prevalence observed in 2004 in phase I and 2010 and 2012 in phase II. These strains formed three clusters, with one cluster containing both phase I and II strains while the other two clusters containing only phase II strains. Despite the distinct clustering, P[4] strains showed a high sequence similarity of 95–100% at the nucleotide level.

### Phylogenetic placement of Kilifi strains in the global context

**Figure 3.**
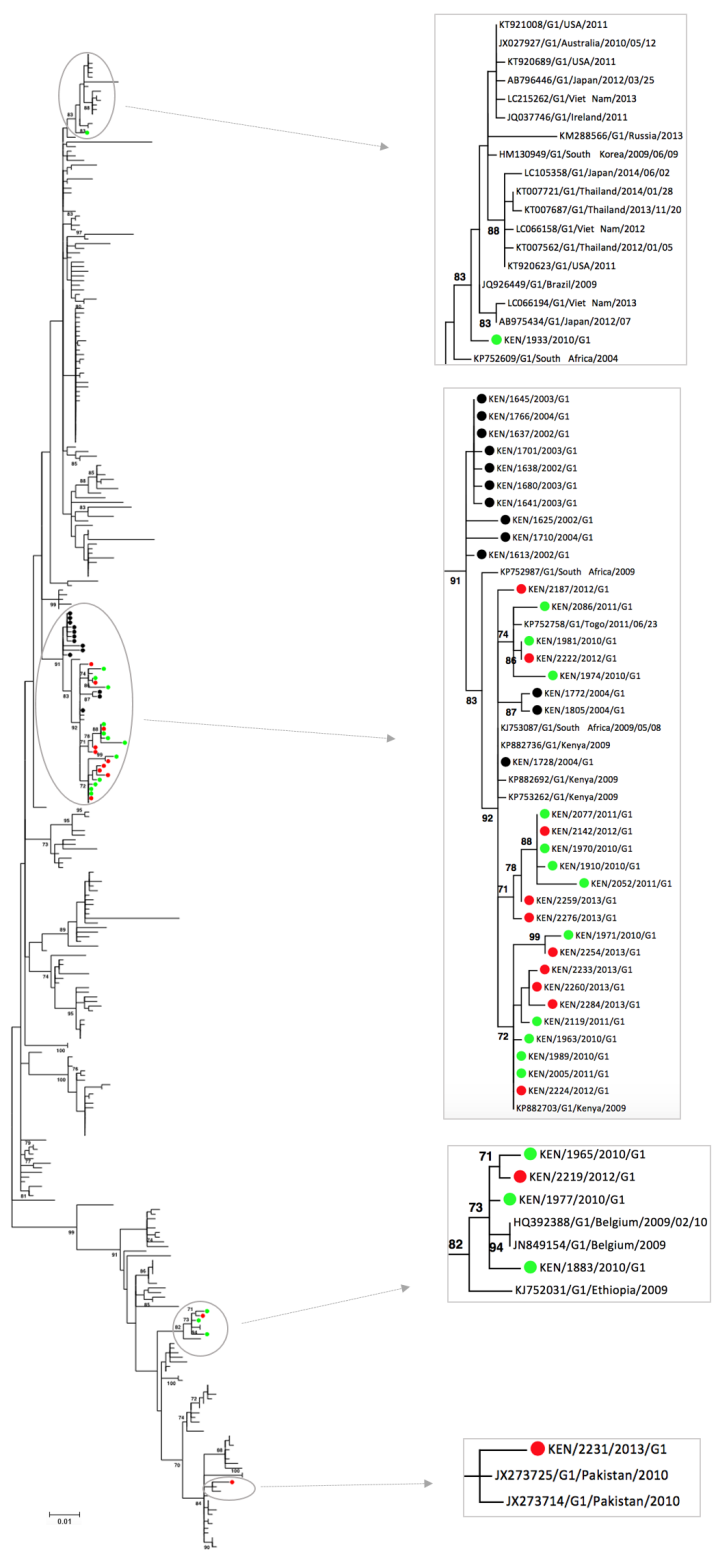
Maximum likelihood tree for VP7 G1 genotype showing the relationship between G1 genotypes from viruses detected in childhood admissions to KCH, Kenya, and to other global G1 genotypes detected between 2002 and 2013. Tree clusters (branches) including Kilifi strains are shown in the expanded boxes. Taxa for Kilifi strains are stratified in three groups, black for 2002–2004, green for 2010–2011 and red for 2012–2013. Only bootstrap values ≥70% are shown. The scale bars indicate nucleotide substitution per site.

**Figure 4.**
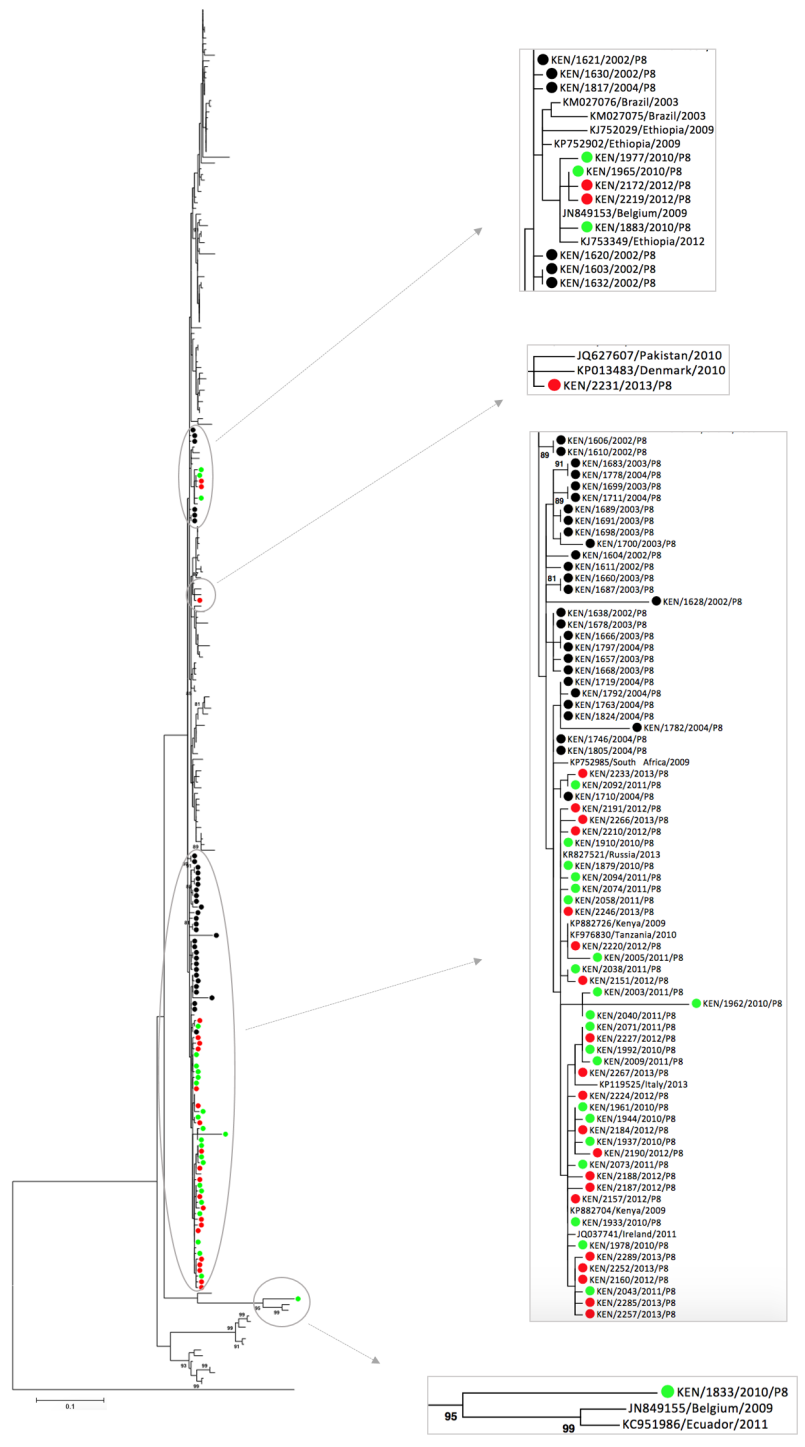
Maximum likelihood tree for VP4 P[8] genotype showing the relationship between P[8] genotypes detected in childhood admissions to KCH, Kenya, and to other global P[8] genotypes detected between 2002–2013. Tree clusters (branches) including Kilifi strains are shown in the expanded boxes. Taxa for Kilifi strains are stratified in three groups, black for 2002–2004, green for 2010–2011 and red for 2012–2013. Only bootstrap values ≥70% are shown. Scale bar represents nucleotide substitutions per site.

**Figure 5.**
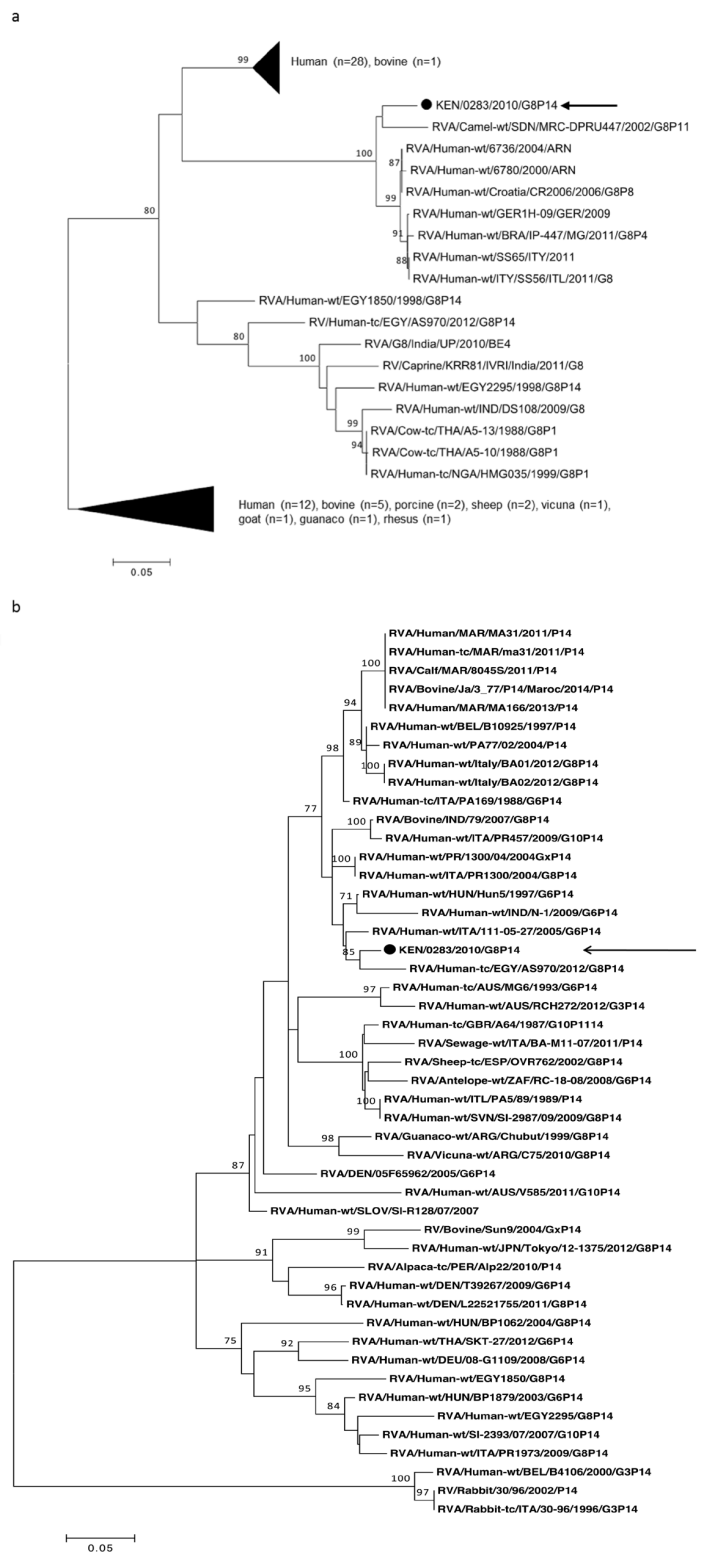
Maximum likelihood phylogenetic tree showing the relationship of the rare G8P[14] strain detected in a child admitted to KCH, Kenya, and, other similar strains detected in humans and animals retrieved from GenBank. ( **A**) shows the phylogenetic relationship of the VP7 G8 genotype to other G8 genotypes. ( **B**) Phylogenetic relationship of the VP4 P[14] genotype to other P[14] genotypes. Sequences for the strains identified in this study are marked by the black filled circle and the arrows. Only bootstrap values ≥70% are shown. Scale bar represents nucleotide substitution per site.

The placement of Kilifi strains in the global context is shown in Figure 3 and Figure 4. Tree clusters leading to Kilifi strains are shown in the expanded boxes. A majority (85%) of the observed G1 strains, clustered away from the other global strains, clustering closely to strains detected in Africa, specifically Kenya, South Africa and Togo. The second cluster comprised only strains from 2010–2012 which distinctively clustered with strains from Belgium and Ethiopia. The last clusters which had only single strains grouped together with strains from Japan and Pakistan. The Kilifi P[8], ( Figure 5) strains were placed into four clusters, where the largest group comprised of Kilifi strains observed in both phase I and II, with external strains observed in Kenya, S. Africa, Tanzania, Ireland and Russia. The second cluster included Kilifi strains from phase I and II with strains from Belgium, Brazil and Ethiopia. The last minor clusters, each made of a single virus, showed a close similarity to strains isolated in Pakistan, Denmark, Ecuador and Belgium.

### Phylogenetic analysis of the rare G8P[14] strain

Whilst several rare GP combinations (not commonly detected) were observed during the study period, G8 associated with a P[14] genotype has overall been rarely detected in human population ^27^. Additionally, there has been an increasing number of human P[14] rotavirus strains globally, which are associated with rabbits, cattle, sheep and guanacos ^28^. We therefore sought to investigate the probable origin of the observed G8P[14] detected in a 14 months old infant in 2010. All cognate sequences for genotypes G8 (n=71) and P[14] (n=47) isolated by 2013 were retrieved from GenBank and phylogenetically compared to the observed genotypes. Duplicate sequences from strains isolated from the same country were removed. The G type in this samples (G8) clustered closely to other G8 strains isolated from humans with a nucleotide and amino acid (aa) identity of 95% and 99%, respectively, and G8 strains isolated from camel showing a nucleotide and aa identity of 94% and 98%, respectively ( Figure 5A). The P[14] genotype showed a high sequence similarity to other P[14] strains isolated from humans and bovine with a nucleotide similarity of 96% and 93%, respectively and aa identity of 98% ( Figure 5B).

## Discussion

This study describes the molecular epidemiology of pre-vaccine RVA strains that circulated in Kilifi, Coastal Kenya from 2002–2004 and 2010–2013. The study spans over a decade in the period before introduction of the nationwide routine RVA vaccination programme in Kenya. The work builds on a previous study ^19^ analyzing strains collected between 2002–2004 (phase I) which highlighted the importance of genotypes G1, G8 and G9 in sub-Saharan Africa during the study period. In phase I surveillance period, genotype-specific primers were used to characterize the strains into different G and P genotypes and this strategy allowed the detection of mixed genotypes in some samples. In the present analysis, a fraction of phase I (46%) and all phase II RVA samples were sequenced, assigned to GP as per the guidelines of the Rotavirus Classification Working Group ^24^ and subjected to phylogenetic analysis.

The strains G1P[8], G9P[8], G8P[4] and G2P[4] were the most common RVA strains in this study, accounting for over 70% of the infections. These strains have also been observed in studies conducted elsewhere in Kenya ^29– 31^ and the world ^32, 33^. Genotype G8P[4] was the third most important strain after G1P[8] and G9P[8] accounting for 15% of RVA infections. The G8 genotype is mostly found in combination with P[4], P[6] and P[8] VP4 specificities ^34^. In this study, the majority (83%) of the G8 strains combined with P[4] types, while only 16% combined with P[8] types. The increase in prevalence of this strain in phase II supports the notion of G8 strain regarded as an unusual and newly emerging strain in the world ^29, 31^. Genotype G3P[8] is also among the common genotypes causing infections in children, and is the second most important strain in Africa and fifth most important globally ^32^. Here, G3P[8] was detected at a low frequency, accounting for only 1% of all the cases. Genotype G12 detection has increased in Africa and has also been observed in Kenya and for the first time in Kilifi during the phase II period (2010–2013).

The detection of atypical GP combinations; G1P[4, 6], G2P[6], G3P[4,6], G10P[8], and G8P[14], albeit at low levels, raised interest in their origins. Despite such atypical strains being less frequent, strains G3P[4] and G2P[6] were found to be the most important causes of diarrhoea in the late 1990s in Ghana ^35^. Genotype G10 has long been reported to infect calves, pigs or cattle but recently has sporadically been reported in humans in several studies ^36, 37^. Similarly strain G8P[14] has recently been detected in humans and is thought to have originated from animals ^27, 38^. In this study, the close association of the observed strain G8P[14] with strains from both humans and animal origins shows a possibility of zoonotic transmission. The increase in diversity of RVA in this setting could be attributed to the emergence of such unusual strains which might have arisen due to zoonotic transmission or re-assortment cases within and between RVA genotypes.

Post-vaccine surveillance studies have reported shifts in the prevalence of RVA genotypes. For instance, data from the USA depicted an increase in prevalence of G3P[8] and G12P[8] in post-vaccine introduction era relative to G1P[8] in the pre-vaccine period ^39^. In contrast, surveillance studies in Australia ^40^ and Belgium ^11^ revealed the dominance of G2 strains in post-vaccine period, relative to G1P[8] in pre-vaccine period. Similar studies in Ghana reported an increase in prevalence of G12P[8] and G10P[6] in the post-vaccine era ^41^. This shift in distribution of genotypes from pre-vaccine introduction to post-vaccine introduction might be associated with either selective vaccine pressure or the natural fluctuations of RVAs. The evidence to support either of these two potential explanations is currently weak. The emergence of uncommon genotypes and increased prevalence of non-vaccine strains warrants close monitoring to determine their circulation in the post-vaccine introduction period and their probable effect on performance of the vaccine.

Overall, the observed strains showed a high nucleotide sequence homology of up to 100%, as observed in the different genotypes. The close genetic relationship of strains observed in phase I and phase II suggest a persistence in circulation of these RVA strains to continuously cause the observed epidemics. In addition, the exclusive clustering of majority of Kilifi strains from the global strains shows that theses strains might have been localized in Kilifi over a long period of time. However, few strains that formed three distinct clusters in both G1 and P[8] global trees, supports the notion of separate introductions and persistence of possibly foreign strains in this setting. Although cases of re-assortment and possible introductions is evident, partial data from only two genes is insufficient in providing a complete understanding of the genetic diversity of such common and not common genotypes. Full genome sequencing will thus illuminate on the complete genomic constellations of these strains and provide data on their evolutionary dynamics. The marked seasonal and longer-term changes in genotype distribution observed in this pre-vaccine surveillance should be considered when interpreting changes to genotype patterns that may follow the introduction of rotavirus vaccine in any setting.

This study had several limitations, e.g. firstly, the exclusive use of di-deoxy sequencing method to genotype phase II samples curtailed our ability to detect mixed infections. During phase I, we identified samples from patients who were infected by more than one RVA genotype since we had used the primer-based methods for genotyping. Di-deoxy sequencing identifies only the dominant genotype in mixed infections resulting to one genotype. The sequencing chromatograms of samples identified as mixed infections in phase I, appeared clean and mono-infected, with no background indicators of co-infections. Secondly, the classification of the strains into lineages and sub-lineages was limited due to the short consensus sequences, since only ~23% and ~67% of the VP4 and VP7 genes were sequenced, respectively. Thirdly, it was not possible to perform comparative analysis of the rare genotype G29 due to unavailability of cognate sequences in GenBank. Only a single reference sequence for genotype G29 had been deposited in GenBank by the time of this analysis.

In conclusion, this study shows that most of the pre-vaccine RVA infections and epidemics have been caused by a diverse range of RVA strains which fluctuated in prevalence from season to season, with some persistent in circulation for a long period. Additionally, new strains might have been introduced in this population and contributed significantly to the epidemics experienced in the pre-vaccine period. The recommendation by WHO for countries to vaccinate infants against rotavirus infection led to the inclusion of Rotarix ^TM^ vaccine in the childhood immunization programme in Kenya. In addition to reducing hospitalization caused by RVA diarrhoea, the vaccine has been reported to offer protection against both homotypic and heterotypic RVA strains ^9^. With the increase in diversity of circulating strains and emergence of rare strains in Kilifi, continuous monitoring will help evaluate the performance of this vaccine against the circulating strains.

## Data availability

The replication data and analysis data for this manuscript are available from the Harvard Dataverse: https://doi.org/10.7910/DVN/LVGYYW ^26^.

Owing to data personal protection concerns, these data are restricted, but will be made available to researchers who meet the criteria for access to confidential data. Details of the criteria for sharing data and the conditions under which data are made available can be found in the KEMRI-Wellcome data sharing guidelines. Users who wish to use the data should send a request to the KEMRI Wellcome Trust Research Programme data governance committee, which can be contacted by emailing: dgc@kemri-wellcome.org.

### Nucleotide sequence accession numbers

Partial sequences for the VP7 and VP4 genes reported in this work were deposited in the GenBank database under the sequential accession numbers MH402005-MH402781 and MH402782-MH403560 for the VP7 and VP4 genes, respectively.

## Supplementary material

**Supplementary Figure 1**. **Pie charts showing proportions of the most common genotypes circulating in Kilifi between 2002–2004 and 2010–2013.** (a) Proportions of the most common VP7 G genotypes. (b) Proportions of the most common VP4 P genotypes. (c) Proportions of the most common G/P combinations. The colour coding for each specific genotype is shown below the pie charts. The others category represents genotypes that circulated in low proportions. This included G3, 10, G12, G29 for panel a, P[6] and P[14] for panel b and G1P[4], G2P[8], G3P[4,6,8], G8P[6,8,14], G9P[4,6], G12P[4,6,8] for panel c.

**Supplementary Figure 2**. **Pairwise distances within the common genotypes indicating the level of identity within genotypes.** The y axis indicates genetic distances determined in MEGA v7 by calculating the proportions of pairwise nucleotide differences between sequences.

**Supplementary Table 1. Evolutionary models used in construction of phylogenetic trees for Kilifi sequences and both Kilifi and Global sequences.** The models were tested using the maximum likelihood method in MEGA v7.

**Supplementary File 1. Accession numbers of VP7 G1 global sequences (list 1) and VP4 P[8] global sequences (list 2) used in phylogenetic comparison with the local strains.**
