# Supplementary figures and images for: Molecular characterization of rotavirus group A strains circulating prior to vaccine introduction in rural coastal Kenya, 2002-2013
Source: Wellcome Open Res. 2019 May 15;3:150. Originally published 2018 Nov 28. [Version 2] doi: 10.12688/wellcomeopenres.14908.2 (PMC6464063; doi:10.12688/wellcomeopenres.14908.2)

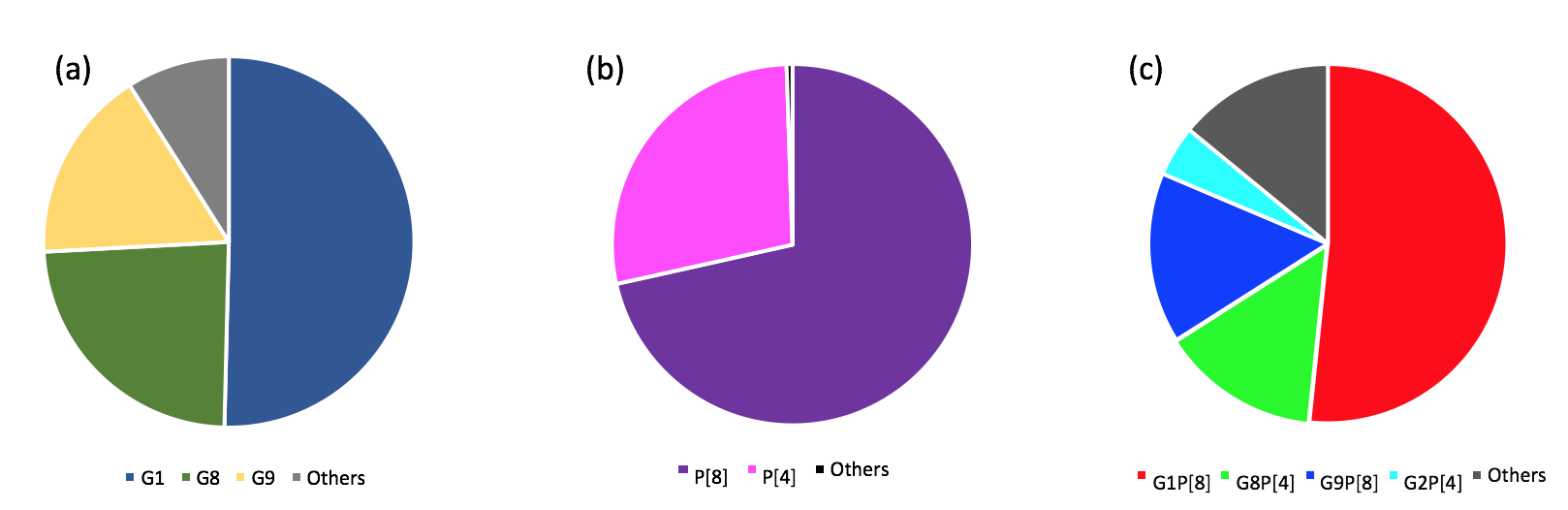

Supplement: Supplementary file 1 [file wellcomeopenres-3-16588-s0000.tgz › 7e976301-fdb6-4c22-8449-36e0f6f04dcf_Supp_fig_1.tif]

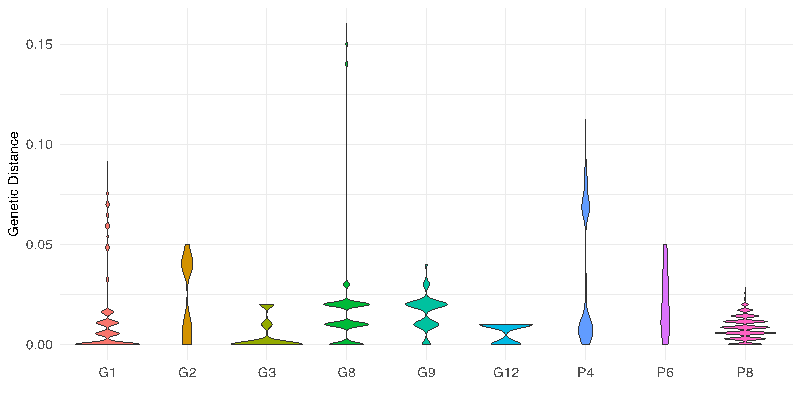

Supplement: Supplementary file 2 [file wellcomeopenres-3-16588-s0001.tgz › da6b2c78-c315-4ca6-969a-8429e970194b_Supp_fig_2.tif]
