# Supplementary material for: Molecular characterization of rotavirus group A strains circulating prior to vaccine introduction in rural coastal Kenya, 2002-2013
Source: Wellcome Open Res. 2019 May 15;3:150. Originally published 2018 Nov 28. [Version 2] doi: 10.12688/wellcomeopenres.14908.2 (PMC6464063; doi:10.12688/wellcomeopenres.14908.2)
Supplement: Supplementary file 3 [file wellcomeopenres-3-16588-s0002.tgz › 3653dfdc-70ec-4255-9013-8e53699e1349_Supp_table_1.docx]

**Supplementary Table 1.**

Evolutionary models used in construction of phylogenetic trees for Kilifi sequences and both Kilifi and Global sequences. The models were tested using the maximum likelihood method in MEGA v7.

| **Genotype** | **Evolutionary Model Used** | |
| --- | --- | --- |
|  | **Kilifi Sequences** | **Kilifi and Global Sequences** |
| G1 | Tamura 3-parameter + invariant | Tamura-Nei distance + gamma |
| G2 | Tamura 3-parameter | NA |
| G3 | Tamura 3-parameter | NA |
| G8 | Tamura 3-parameter + invariant | NA |
| P4 | Tamura 3-parameter + gamma | NA |
| P8 | Tamura-3-parameter + I | Tamura-3-parameter + G4 |
