# Supplementary material for: Molecular characterization of rotavirus group A strains circulating prior to vaccine introduction in rural coastal Kenya, 2002-2013
Source: Wellcome Open Res. 2019 May 15;3:150. Originally published 2018 Nov 28. [Version 2] doi: 10.12688/wellcomeopenres.14908.2 (PMC6464063; doi:10.12688/wellcomeopenres.14908.2)
Supplement: Supplementary file 4 [file wellcomeopenres-3-16588-s0003.tgz › 476aec34-1d37-4cd0-a723-1ebdfa1174af_Supplementary_File_1.docx]

**Supplementary List 1. Accession numbers of VP7 G1 global sequences used in phylogenetic comparison with the local strains.**

AB561878,KJ753152,KX638511,JN192123,KC443493,KJ752155,KP222808,KP222847,KT920722,JX029851,MF184811,KP222820,KM027243,KX638527,JN869255,KU360938,MF161694,AB905455,GQ996880,KJ432642,KJ432658,AB975434,LC066194,JQ926449,JX027927,KT921008,KM247270,KT920689,KT920623,HG917364,KJ412737,JQ069520,JF490088,KT920898,MF168306,MF184781,JQ926446,JX683594,JN192096,KP752609,HQ392453,JN258941,JN849136,JQ037742,JQ037751,KF414588,KJ432654,KJ432665,KJ919860,KP645283,MF494737,JQ069534,JX029852,KF414539,KF414548,KJ919845,KJ919876,KF202504,KF202507,KF006910,HQ392461,JN258908,KF414538,KJ432646,KJ919886,MF494738,EF690736,DQ492674,KX638530,GQ452916,JQ069536,JX154488,KP753217,KJ751718,KP752653,KP752653,KJ752042,KJ752042,KP883143,KP882505,KM660406,JX470510,KX638552,KX517810,KJ432638,MF161791,KC510182,KF356358,KF636217,KP216561,KJ870858,KC223598,MF161707,KM027239,KM027242,JF490143,FJ948838,JF968588,KX638516,JN258917,KJ919828,JN192105,KP753022,JQ069528,KF636173,KJ752267,KP752676,KJ432639,KJ752243,KJ752243,KP222814,MG181452,HQ392381,GQ996873,JF490517,KU973947,JX027864,GU979204,KP735839,HM130937,HM130943,JX027884,JN706571,KM027236,KU243671,JF490135,JN258879,JQ710674,JX458967,KY972035,JN232069,MG181364,KX632347,JF968570,KM027200,JX027850,KU973951,KP752904,DQ857949,KP752987,KJ753087,KP882736,KP882692,KP753262,KP752758,KP752758,AB975421,AB975432,KT007708,KJ412625,KT948654,LC105000,KP007159,GQ452918,GU377173,KF006870,KX467662,KM068245,KF812574,KU243667,KM276825,KM288566,FJ435209,KF006882,GQ452914,FJ919246,JX027905,MF168131,HQ537514,FJ811903,JQ069533,LC018668,JF490812,JF490824,JF490275,JF490539,JN706233,KC762221,JN706264,KF006923,JN849150,KJ751751,KJ751938,KJ753231,KJ753754,JX273714,JX273725,KY497502,KP013534,GU377133,KJ752546,GU979202,KJ919912,HQ392388,JN849154,KJ752031,GU979206,JX683600,JX943614,HQ392415,HG917362,JF490385,JN232070,KY039372,KJ626790,JN706202,MF161670,MF161693

**Supplementary List 2. Accession numbers of VP4 P[8] global sequences used in phylogenetic comparison with the local strains.**

JF748715,KJ432807,JQ037752,KJ135220,KM230939,KF614647,HQ451950,KJ432842,GU226775,KP013489,KJ432857,KF614643,GU226776,KX228860,JN705952,JX645700,KT369151,KJ432802,KJ432848,HQ451965,KP862856,KX545300,KX702212,KJ752861,KP119521,KM205777,KJ432818,KJ432846,KJ432804,KX702211,KP013496,JX273746,KP119542,HQ451964,KF614649,KJ432839,KM027073,JX446613,EU045232,KP013477,KF202511,KJ432855,KX646589,KJ752342,KT387269,KJ751560,KX646557,KJ921617,KX646604,KT988252,KJ753471,LC066148,JN706068,JN400033,KM027095,JN706522,KX646555,KX646559,KM027088,JQ639399,KX702208,JQ639403,KX646558,KX646576,AB905377,KX646569,LC066192,KJ768415,KF414575,JX195077,KC890874,KJ751749,KM027089,JN849153,KJ753349,KJ752029,KU174069,KJ560524,KT921006,KU174068,KP119522,KJ768419,KU174075,JN706015,JN705964,JN705995,KT369154,KT369155,KU174080,JX437057,KR827520,KU174089,KP013474,JQ037741,KR827521,KP882704,KP882737,KP753260,KP882693,JN605451,KP882726,KP752985,KF976830,KF976815,KP119525,KP119531,KP222875,KY056539,KP013494,KF414620,KP013488,KF614631,KF614629,KU174085,JN706520,KM027114,KM027116,KU174094,KU174088,JQ693569,KF614648,KF614645,KM008694,KU174087,KR181904,KU174095,KU174102,KT988164,JQ627600,JN695617,JX273730,KX646599,KX646601,AB905373,KX646603,AB905374,KP222865,KM379148,KM379147,KU174096,KJ432809,KM027092,EU045237,KJ432786,KY616896,JF990831,EU045233,KM027070,HM245026,KU174086,JN849155,KC951986,KC951993
