# Supplementary material for: Molecular characterization of rotavirus group A strains circulating prior to vaccine introduction in rural coastal Kenya, 2002-2013
Source: Wellcome Open Res. 2019 May 15;3:150. Originally published 2018 Nov 28. [Version 2] doi: 10.12688/wellcomeopenres.14908.2 (PMC6464063; doi:10.12688/wellcomeopenres.14908.2)
Supplement: Peer review history

# Peer review: Reviewer response for version 1

**DOI:** 10.21956/wellcomeopenres.16252.r34548

**Published:** 2019-04-10

**Authors:** Martin Munene Nyaga

**Affiliations:** 1 Next Generation Sequencing Unit, Department of Medical Virology, Faculty of Health Sciences, University of the Free State, Bloemfontein, South Africa

This is the first detailed study on the prevalence of RVA genotypes causing diarrhoea in children in rural coastal Kenya spanning over a decade to investigate the evolutionary dynamics of human rotaviruses pre-vaccine introduction, which present original findings as per the problem statement of the study.

 While this reviewer finds the study appropriate for indexing, we do suggest the authors should consider the following suggestions before final acceptance as noted by the reviewer:

**Introduction:**

1. The authors need to clarify that the dominance of G2P[4]s in Belgium and Brazil were not linked to rotavirus vaccine pressure. Recent data in different parts of the world indicate that the vaccine provides heterotypic immunity against a variety of rotavirus strains including G2P[4]s. Furthermore, G2P[4]s are known to have a cyclic trend where they disappear and reappear in four-year cycles which could have been the case at the time.
2. Encodes 11 proteins, sometimes 12 (NSP5/6).
3. Consider rephrasing to “G1-4, G9, and G12 in combination with P,[4], P[6] and P[8] are the most common genotypes…”
4. 37P types and 27 G types have been described globally. Another 13P and 7 G types were proposed as of 2018 and await rectification by the RCWG and ICTV.

**Methods:**

1. Indicate the ethics number as approved by SERU.

**Results:**

1. Add a comma before the word ‘respectively’ throughout the document.
2. Uncommon strains bearing G1P[4]…
3. How many sequence-based methods were applied? To my understanding only Sanger sequencing, right? If you used NGS you could have detected the mixed infections. Rectify accordingly.
4. Homologous meaning completely similar. “High homology” is not scientifically sound. Again 92% is very diverse for different genotypes. Check proposed cut off values.
5. There are partial sequences (Incomplete CDs) but there is no partial sequencing method unless if refereeing to primer based sequencing based on targeted regions.

I have read this submission. I believe that I have an appropriate level of expertise to confirm that it is of an acceptable scientific standard.

# Response

**Published:** 2019-05-06

**Authors:** Mike Mwanga

**Affiliations:** KEMRI Wellcome Trust Reseach Program, Kenya

*The authors need to clarify that the dominance of G2P[4]s in Belgium and Brazil were not linked to rotavirus vaccine pressure. Recent data in different parts of the world indicate that the vaccine provides heterotypic immunity against a variety of rotavirus strains including G2P[4]s. Furthermore, G2P[4]s are known to have a cyclic trend where they disappear and reappear in four-year cycles which could have been the case at the time.*

**Our response:** This has been revised in the updated version. (Refer to the Introduction section).

*Encodes 11 proteins, sometimes 12 (NSP5/6),*

**Our response:** This has been revised in the updated version. (Refer to the Introduction section). 

*Consider rephrasing to “G1-4, G9, and G12 in combination with P,[4], P[6] and P[8] are the most common genotypes…”*

**Our response:** We also received similar comments from the other reviewers and this sentence has since been revised.

*37P types and 27 G types have been described globally. Another 13P and 7 G types were proposed as of 2018 and await rectification by the RCWG and ICTV.*

**Our response:** This has been revised in the updated version.

***Methods:***

*Indicate the ethics number as approved by SERU.*

**Our response:** The SERU number is now indicated. 

***Results:***

*Add a comma before the word ‘respectively’ throughout the document.*

**Our response:** This has been checked and corrected throughout the manuscript.

*Uncommon strains bearing G1P[4]..*

**Our response:** This has been rectified in the Results section.

*How many sequence-based methods were applied? To my understanding only Sanger sequencing, right? If you used NGS you could have detected the mixed infections. Rectify accordingly.*

**Our response:** One sequencing method, i.e, di-deoxy sequencing was used in this study. Mixed infections identified in phase I (2002-2004) were determined by genotype-specific primer-based PCR method and previously published (Nokes *et al.*, 2010) This has been made clear in our revised version.

*Homologous meaning completely similar. “High homology” is not scientifically sound. Again 92% is very diverse for different genotypes. Check proposed cut off values.*

**Our response:** **This has been  checked and reworded to read “with sequence similarities of between 92-100% at nt level”. (Refer to the Results section).

*There are partial sequences (Incomplete CDs) but there is no partial sequencing method unless if refereeing to primer-based sequencing based on targeted regions.*

**Our response:** This has been changed from “ **partial sequencing method”** to “ **di-deoxy sequencing method” **(Refer to the Discussion section).

# Peer review: Reviewer response for version 1

**DOI:** 10.21956/wellcomeopenres.16252.r34355

**Published:** 2019-02-06

**Authors:** Mathew D Esona

**Affiliations:** 1 Division of Viral Diseases, Centers for Disease Control and Prevention, Atlanta, GA, USA

**Summary of article**:

 This article by Owor et al., described the genotypes circulating pre-vaccination in Kilifi, Kenya. They reported G1P[8] as the most predominant strain in circulation during the study periods.

 1) Is the work clearly and accurately presented and does it cite the current literature? With the exception of citing genotype G12P[8] as the sixth most common genotype in circulation, the authors have used recent and current citations. I would recommend that authors edit the statement in paragraph 3 to include G12P[8].

 2) Authors use sequencing method to determine these genotypes. With sequencing you can not call mixed genotypes. Authors should explain how mixed genotypes where determined and confirmed by sequencing reaction.

 3). The above data adds very little to the information we have on rotavirus genotypes circulating in Kenya. I recommend that authors take this one step further, by performing a next generation sequencing on selected genotypes. This will give the public more information on the interaction between the vaccine strains and the wild type strains in Kenya.

I have read this submission. I believe that I have an appropriate level of expertise to confirm that it is of an acceptable scientific standard, however I have significant reservations, as outlined above.

# Response

**Published:** 2019-04-09

**Authors:** Mike Mwanga

**Affiliations:** KEMRI Wellcome Trust Reseach Program, Kenya

- **Reviewer comment:** Is the work clearly and accurately presented and does it cite the current literature? *With the exception of citing genotype G12P[8] as the sixth most common genotype in circulation, the authors have used recent and current citations. I would recommend that authors edit the statement in paragraph 3 to include G12P[8].*
- **Our response:** We appreciate this correction and have modified the sentence (starting line #31) to read "Globally, G1P[8], G2P[4], G3P[8], G4P[8] and G9P[8] have been identified as the most common genotypes (in decreasing order) while G12P[6] and G12P[8] have recently been reported as emerging genotypes.

 

- ***Reviewer comment: **Authors use a sequencing method to determine these genotypes. With sequencing, you cannot call mixed genotypes. Authors should explain how mixed genotypes were determined and confirmed by sequencing reaction.*
- **Our response:** We agree that our sequencing strategy could not support calling mixed infections. This is a limitation we highlight in the discussion section of the manuscript. As explained in the manuscript, the study was divided into two phases (I & II). In phase I (2002-2014), genotyping was previously done using the PCR primer-based strategy https://www.ncbi.nlm.nih.gov/pmc/articles/PMC2923076/. Phase II of the study used sequencing to genotype. We have made this clear throughout the manuscript in this revised version.

 

- ***Reviewer comment: **The above data adds very little to the information we have on rotavirus genotypes circulating in Kenya.*
- **Our response**: We point out that our RVA genotypic and sequence data from Kenya represents a large dataset in the period leading up to the vaccine introduction that spans a wide time period. We feel this offers additional information than previously available by which to explore patterns in one part of the country in a period spanning a decade to show natural fluctuations and the extent of genetic diversity in circulating strains in the absence of a vaccination programme. We further examined the global context of the local strains to understand the nature of their source year-in-year-out. Additionally, to the best of our knowledge, this is the first study in Kenya, to generate vast rotavirus partial seqence data from a large sample set collected before vaccine introduction, hence an addition to the limited sequence data for tracking RVA transmission. We believe that this study provides an important baseline for future studies especially those attempting to interpret post-vaccine introduction strain patterns.

 

- ***Reviewer comment;*** *I recommend that authors take this one step further, by performing a next-generation sequencing on selected genotypes. This will give the public more information on the interaction between the vaccine strains and the wild type strains in Kenya.*
- **Our response**: We thank the reviewer for this suggestion. We are developing an in-house NGS protocol but the results will form the basis of a separate publication.

​​​

# Response

**Published:** 2019-05-06

**Authors:** Mike Mwanga

**Affiliations:** KEMRI Wellcome Trust Reseach Program, Kenya

Is the work clearly and accurately presented and does it cite the current literature?

**Reviewer comment:** *With the exception of citing genotype G12P[8] as the sixth most common genotype in circulation, the authors have used recent and current citations. I would recommend that authors edit the statement in paragraph 3 to include G12P[8].*

**Our response:** We appreciate this correction and have modified the sentence to read “ Globally, G1P[8], G2P[4], G3P[8], G4P[8] and G9P[8] have been identified as the most common genotypes (in decreasing order) while  G12[P6] and  G12P[8] have recently been reported as emerging genotypes.”

*Authors use sequencing method to determine these genotypes. With sequencing you cannot call mixed genotypes. Authors should explain how mixed genotypes where determined and confirmed by sequencing reaction.*

**Our response**: We agree that our sequencing strategy could not support calling mixed infections. This is a limitation we highlight in the discussion section of the manuscript. As explained in the manuscript, the study was divided into two phases (I & II). In phase I (2002-2014), genotyping was previously done using the PCR primer-based strategy https://www.ncbi.nlm.nih.gov/pmc/articles/PMC2923076/. Phase II of the study used sequencing to genotype. We made this clear through the manuscript in this revised version.

*The above data adds very little to the information we have on rotavirus genotypes circulating in Kenya.*

**Our response**: We appreciate this concern but would also like to point out that RVA genotypic and sequence data from Kenya in the period leading to the vaccine introduction is sparse. We examine and present a reasonably large dataset to explore patterns in one part of the country in a period spanning a decade to show natural fluctuations and the extent of genetic diversity in circulating strains in the absence of a vaccination programme. We further examined the global context of the local strains to understand the nature of their source year-in year-out. We believe that this study provides an important baseline for future studies especially those attempting to interpret post-vaccine introduction strain patterns.

*I recommend that authors take this one step further, by performing a next-generation sequencing on selected genotypes. This will give the public more information on the interaction between the vaccine strains and the wild type strains in Kenya.*

**Our response**: We thank the reviewer for this suggestion and we totally agree with it. We actually have already started generating some sequence data using the NGS approach but are convinced that this will be best presented as a separate report in which the focus will not be genotype prevalence and long-term patterns.

# Peer review: Reviewer response for version 1

**DOI:** 10.21956/wellcomeopenres.16252.r34354

**Published:** 2018-12-21

**Authors:** Ernest Wandera Apondi

**Affiliations:** 1 Kenya Research Station, Institute of Tropical Medicine, KEMRI/Nagasaki University, Nairobi, Kenya

Owor *et al *conducted a longitudinal epidemiological study of rotavirus genotype distribution patterns in Coastal Kenya before the nationwide introduction of rotavirus vaccine. The authors employed methods such as enzyme immunoassay (EIA) for the detection of group A rotavirus (RVA) and partial sequencing of RVA positive samples in VP4 and VP7 segments for G and P genotyping. Data analysis reveals remarkable genetic diversity of RVA strains circulating in this area, characterized by substantial frequencies of unusual, mixed and emerging genotypes. Temporal fluctuation in RVA genotypes was observed, with major shifts in G-P predominance involving G1P[8] and G8P[4].

 The study was well conducted and the manuscript well written. The findings of this study are timely in light of the recent introduction of rotavirus vaccine in Kenya and provide the baseline data necessary for the assessment of vaccine effectiveness. This baseline data will also allow monitoring of RVA G and P genotype changes that may alter vaccine effectiveness or that may be a result of vaccination, such as possible breakthrough events under vaccine immune selective pressure.

 Of noteworthy, a rare G8P[14] strain was detected in this study and the partial sequencing of this strain indicated that its VP7 segment is closely related to humans and animals while its VP4 segment clustered closely to that of human and bovine origin. Due to the unconventional nature of this and many other uncommon strains detected in this study, it will be useful to sequence and characterize the full genomes of the representative strains in order to provide important insights into their evolutionary dynamics.

 Furthermore, since the uncommon strains, such as the ones detected in this study are either partially or fully heterotypic to the currently licensed RVA vaccines (RV1 and RV5), vaccine effectiveness against these strains needs to be closely monitored.

I have read this submission. I believe that I have an appropriate level of expertise to confirm that it is of an acceptable scientific standard.

# Response

**Published:** 2019-04-09

**Authors:** Mike Mwanga

**Affiliations:** KEMRI Wellcome Trust Reseach Program, Kenya

We thank the reviewer for this appraisal of our work. We confirm that we are developing a whole genome sequencing protocol to sequence a select group of positive samples. This will include unusual strains and a longitudinal set spanning before to after introduction of the vaccine. The results will be presented in a separate publication.

# Response

**Published:** 2019-05-06

**Authors:** Mike Mwanga

**Affiliations:** KEMRI Wellcome Trust Reseach Program, Kenya

We thank the reviewer for applauding the work. We can also confirm that whole genome sequencing of the positive samples and including those collected in the post-vaccine introduction period has commenced.
